# Research hot topics and frontiers in social anxiety over the past decade: a CiteSpace bibliometric analysis based on Web of Science database from 2013 to 2023

**DOI:** 10.3389/fpsyt.2024.1421907

**Published:** 2024-10-23

**Authors:** Peng Zhang, Mingliang Wang, Lin Ding, Jianing Zhang, Yuqing Yuan, Xin Tian

**Affiliations:** ^1^ Key Research Base of Humanities and Social Sciences of the Ministry of Education, Academy of Psychology and Behavior, Tianjin Normal University, Tianjin, China; ^2^ Faculty of Psychology, Tianjin Normal University, Tianjin, China; ^3^ Tianjin Key Laboratory of Student Mental Health and Intelligence Assessment, Tianjin Normal University, Tianjin, China; ^4^ School of Foreign Languages, Tianjin Normal University, Tianjin, China

**Keywords:** social anxiety, CiteSpace, bibliometric analysis, data visualization, Web of Science

## Abstract

**Objective:**

To understand the current study of social anxiety (SA) over the past decade, and to analyze the research hot topics and frontiers in this field.

**Methods:**

CiteSpace 6.2.R3 was used to analyze the literature on SA collected in the Web of Science Core Collection database from 2013 to 2023.

**Results:**

A total of 9940 literature were included after the screening, and the annual publication volume showed a steady increase. The results emphasize that Zvolensky MJ, Pine DS, and Heimberg RG are important authors in the field of SA. The United States has the highest number of publications, with the University of California System contributing the most. Research hotspots include cognitive impairment, risk factors, complications, neuroimaging, and intervention strategies. SA related to the “theory of mind”, “bullying victimization”, “mobile phone”, “network analysis”, “technology”, and “satisfaction” are emerging research foci.

**Conclusion:**

This study identifies the current situation and trends of SA research, and provides a reference for future research topics and directions.

## 1 Introduction

Individuals with social anxiety (SA) are characterized by showing intense fear and avoidance in many social situations, and in severe cases can develop a psychiatric disorder (1, 2). SA has become the third most common mental health problem in the world, with the lifetime prevalence of SA in the U.S. as high as 12%, with 90% of cases occurring before the age of 23 (3). A survey of seven countries around the world found that the prevalence of SA in younger age groups (16–29) ranged from 23% to 58% (4). In addition, more demographic information shows that women are more likely to suffer from SA, and exhibit higher levels of anxiety, particularly among those who are unemployed, living in suburban areas, and not completing secondary school education (4, 5). Socially anxious people are socially isolated and have difficulty forming friendships and close relationships. They focus more on themselves, negative and threatening messages, leading to avoidance of social situations thus affecting the individual’s life and social functioning. Additionally, SA can trigger depression and increase the risk of suicidal behavior, substance abuse, and problematic social media use, which can threaten an individual’s physical and mental health (6–8). Given the high prevalence and danger of SA, researchers have become increasingly concerned about this group, and research in the field of SA has progressed greatly in the last decade (8–10).

Although scholars have reviewed SA in recent years, most of the existing studies have explored a single research question or subjectively summarized the main content and research progress in a certain field, focusing on qualitative analysis of the current research progress at the theoretical level, and this kind of review relying on the text to bring us a limited amount of information (11, 12). The etiological model of SA emphasizes the complexity of factors that contribute to its generation and maintenance, including the interplay between individual factors (genetic, biological, cognitive, social skills, etc.) and environmental factors (peer relationships, school environment, life events, parental behavior, culture, etc.). Given the complexity of the causes of SA, it is crucial to situate the study of SA within a more comprehensive framework (13). In addition, the previous bibliometric analysis of SA only focused on specific subject group and country (14, 15), resulting in a lack of systematic and intuitive presentation of the development context, research hotspots, and future research trends of the entire SA research field, which cannot help readers to quickly and efficiently understand the dynamics and development trends of this research field. Bibliometrics, with the help of knowledge mapping, expresses the information of literature through visual graphs, enabling researchers to conduct in-depth searches and extended readings on specific topics of interest. so as to describe, explain, and infer research hotspots within disciplines or knowledge domains (16).

CiteSpace, created by Prof. Chaomei Chen of Drexel University, is one of the most distinctive and influential visualization software in the field of information analysis. According to Prof. Chen, CiteSpace can be applied to a variety of research fields, and is equally valuable in analyzing the social sciences as well as the natural sciences (17). In summary, this study aims to systematically analyze the basic features and research dynamics of SA research in the past decade from a global perspective, providing ideas and inspiration for subsequent research. Specifically, this paper analyzes the number of publications, authors, countries, institutions, and journals using CiteSpace visualization software to obtain an overview of SA, and analyzes the research hotspots and frontiers through keywords and cited references.

## 2 Methods

### 2.1 Analysis methods

The nodes in CiteSpace represent the analyzed objects, and the frequency of their occurrence is reflected by the size of the circle. The timeframe of their occurrence is reflected by the spectrum of the circle. A connection between two nodes indicates the existence of a co-citation between them, and the length and thickness of the connection indicates the strength of the connection between the two nodes. Some nodes are surrounded by purple rings, reflecting the important indicator of centrality, which means the node has extensive connections to nodes in other domains. Such nodes are often hubs of disciplines or knowledge domains, and have special significance in the node network, requiring focused attention and analysis. CiteSpace software is highly acclaimed for its solid theoretical foundations, intuitive graphs, objective and extensive data, and readability, providing a clear summary of the macroscopic overview of a domain.

### 2.2 Data sources

**Figure 1:**
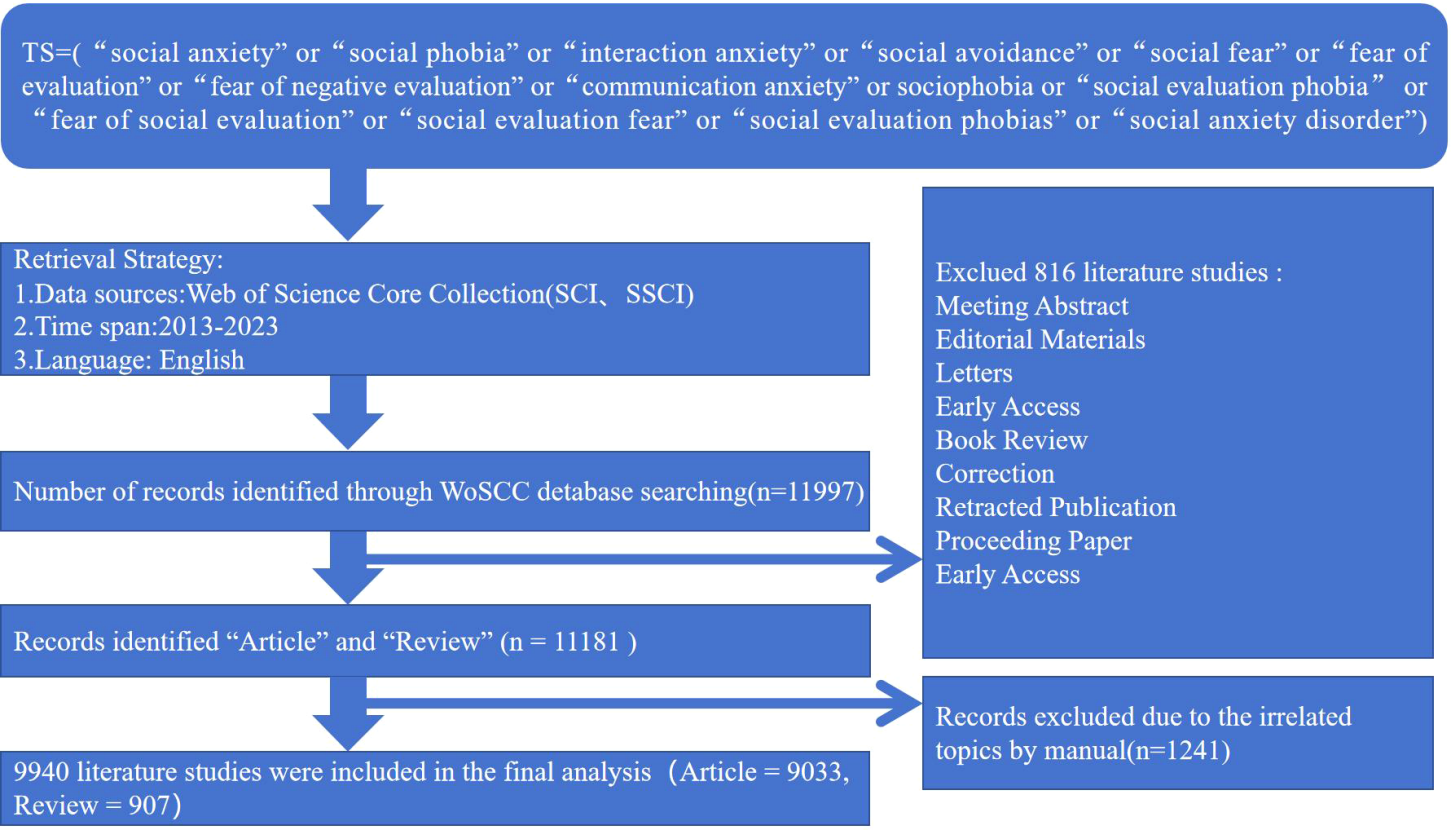
Data retrieval flow chart.

All data were obtained from the Web of Science (WoS) database. WoS is the most commonly used and recognized database for scientific or bibliometric studies because of its broad coverage, data integrity, and high compatibility with CiteSpace (18). The search terms were selected with reference to three meta-analyses (6, 8, 19), combined with the MeSH terminologies. Using the “Advanced Search” function in the WoSCC database, the search query was set as TS=(“social anxiety” or “social phobia” or “interaction anxiety” or “social avoidance” or “social fear” or “fear of evaluation” or “fear of negative evaluation” or “communication anxiety” or sociophobia or “social evaluation phobia” or “fear of social evaluation” or “social evaluation fear” or “social evaluation phobias” or “social anxiety disorder”). The publication date range was set from 2013 to 2023, and the language was limited to English. By excluding non-articles, reviews, and irrelevant literature, a total of 9940 documents were exported. These retrieved documents would be exported in the form of all records and references. CiteSpace was then used to filter and convert the records. We utilized the “Remove Duplicates” function to reevaluate the data. The process of data extraction was presented in **Figure 1**.

## 3 Results

### 3.1 Analysis of the basic profile of SA research

#### 3.1.1 Analysis of annual publications

**Figure 2:**
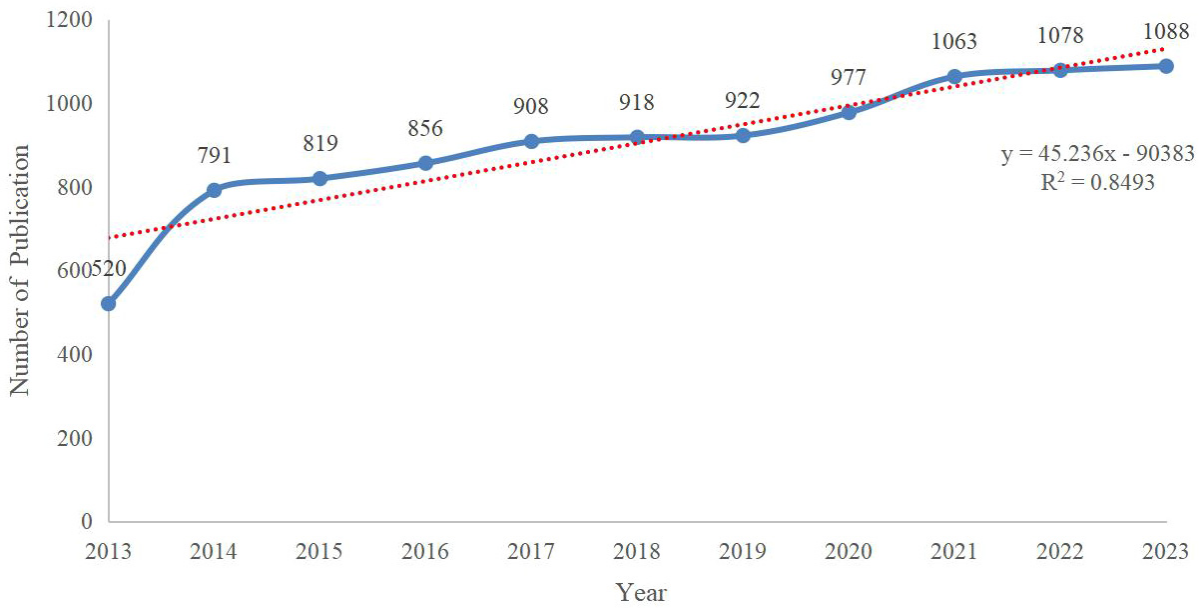
The annual number of publications related to SA.

The number of annual publications is an important indicator of the development of scientific research, which can visualize the changes in the amount of scientific knowledge and the progress of research in a certain research field, thus helping scholars to predict the development of the research situation and trends. We retrieved 9940 publications on SA published from 2013 to 2023. As can be seen in **Figure 2**, the number of studies in the field of SA in the past ten years has shown a continuous growth trend, and the fitting curve of publication numbers can also show that research enthusiasm in this field has not decreased.

**Figure 3:**
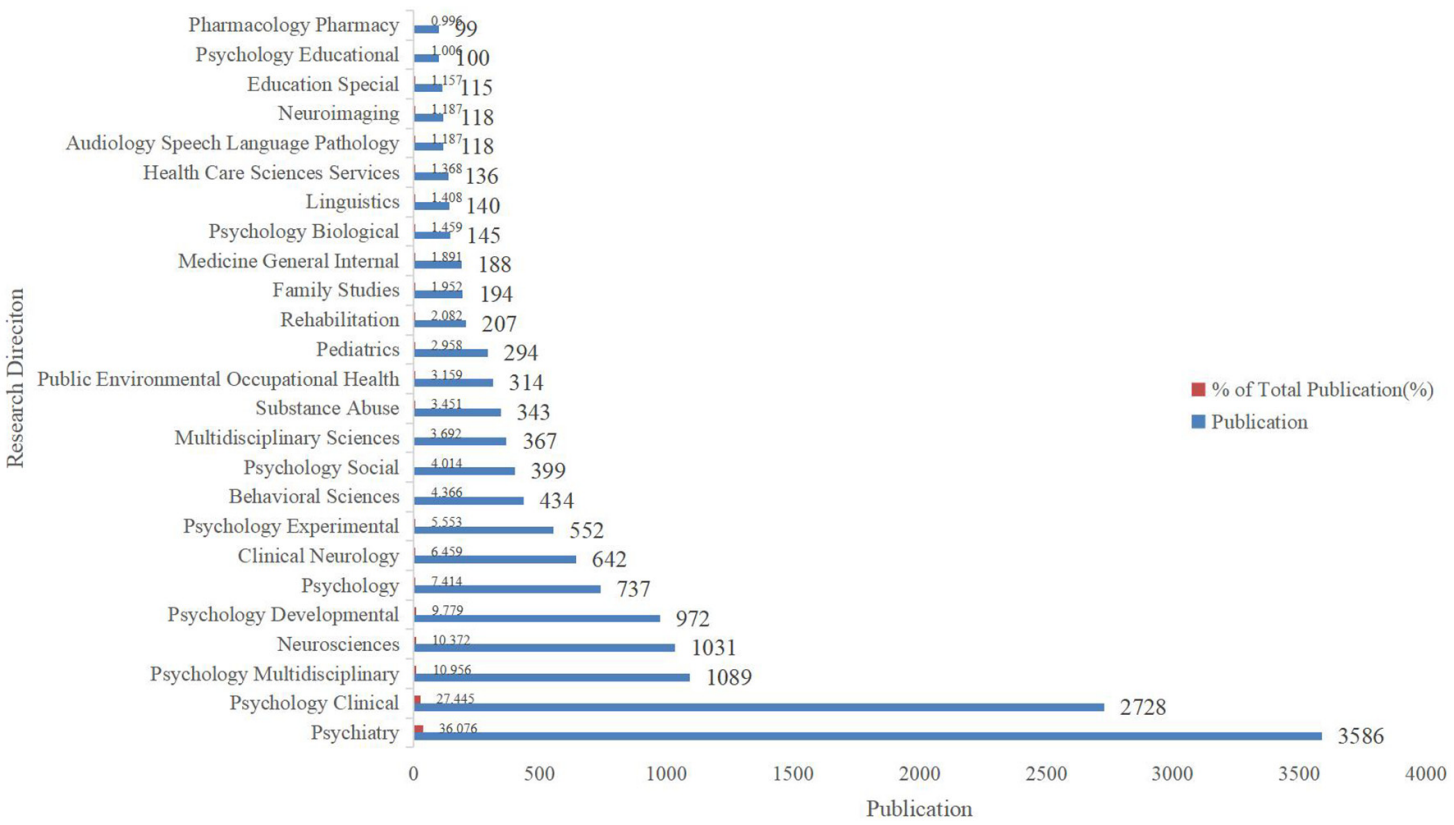
Publications of different research directions on SA.

Specifically, the annual number of publications increased rapidly from 2013 to 2014, stabilized but remained above 800 between 2015 and 2020, and exceeded 1,000 from 2021 to 2023. Therefore, it is evident that a high level of concern and interest in SA continues to be maintained by academics. From the perspective of research orientation (**Figure 3**), the literature on SA is relevant to multiple areas of research, especially psychiatry, psychology, and neurosciences. This shows that researchers have paid attention to this important issue in different disciplinary contexts, reflecting the trend of multi-disciplinary integration in the field of SA.

#### 3.1.2 Analysis of authors

**Figure 4:**
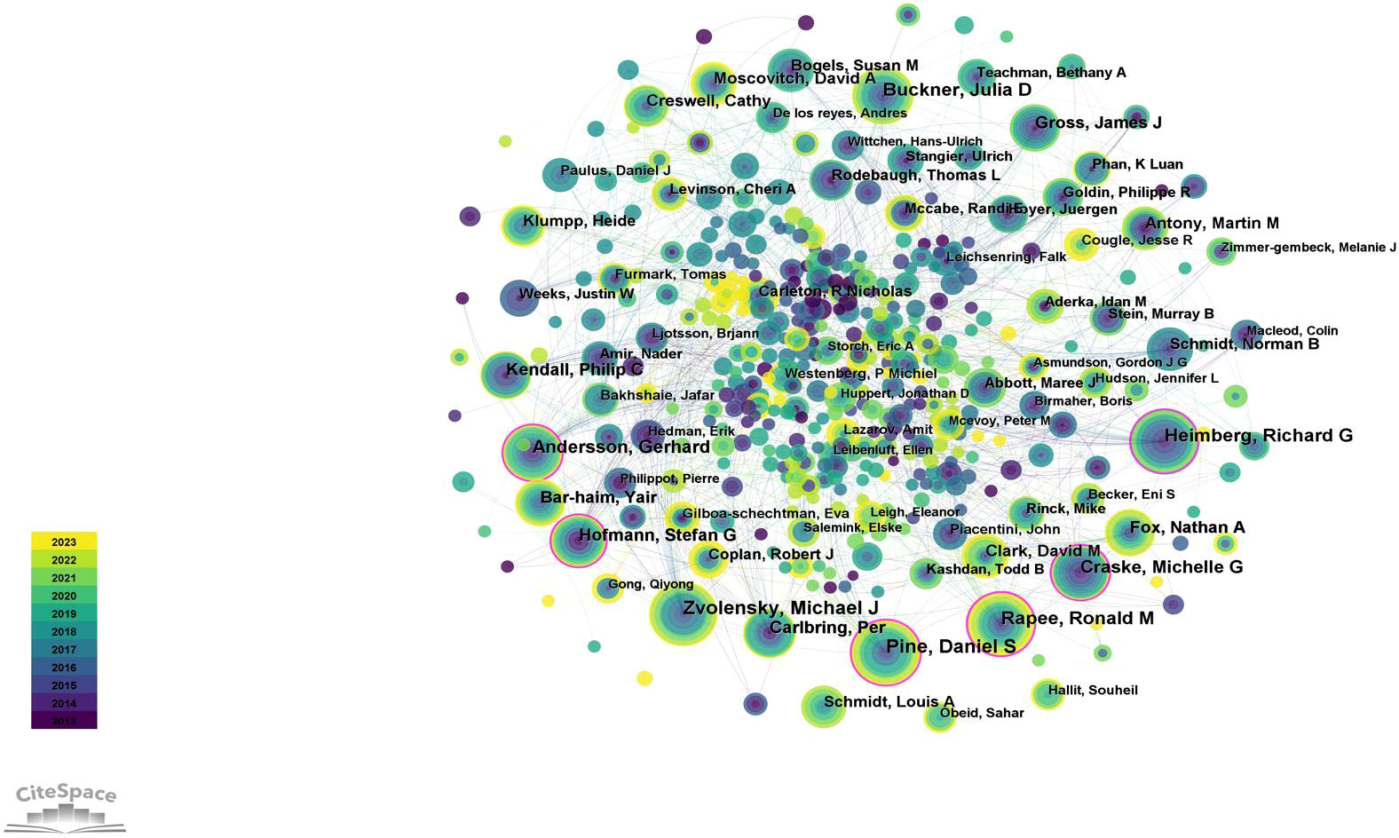
The knowledge map of the author’s cooperation network.

Authors are the most foundational research force, and author analysis helps to provide an overview of the research in the field by identifying prolific authors, influential authors, and the intensity of cooperation among authors. The results of the author’s collaboration network analysis indicated that researchers were more collaborative (**Figure 4**), which will facilitate further development in the field of SA.

The quantity of authors’ literature reflects their knowledge output capacity, while the quality of literature reflects their academic impact. Knowledge output capacity is measured by the number of publications, while academic influence is measured by centrality. In the generated knowledge map, the size of the node represents the frequency of publication, and the purple outer ring of the node represents centrality. The thickness of the purple outer ring indicates centrality: a thicker ring signifies higher centrality. **Table 1** displays the top 10 authors by publications and centrality, identifying the pivotal figures in SA research. We can see that nine authors have both high publications and centrality. They are Zvolensky, Michael J (82,0.06), Pine, Daniel S (81,0.16), Heimberg, Richard G (78,0.11), Rapee, Ronald M (78,0.15), Andersson, Gerhard (68,0.11), Craske, Michelle G (57,0.16), Carlbring, Per (53,0.07), Hofmann, Stefan G (52, 0.15), and Kendall, Philip C (49, 0.07), indicating that these nine authors have made outstanding contributions to the field of SA. Buckner, Julia D ranked fifth in the number of publications and fourteenth in the centrality. Although the number of articles published by De los reyes, Andres is not large, the centrality ranks tenth, indicating that the author still has great potential in the field of SA.

**Table 1:** The top 10 authors related to SA.

Rank	Publications	Centrality	Author	Publications	Centrality	Author
1	82	0.06	Zvolensky, Michael J	81	0.16	Pine, Daniel S
2	81	0.16	Pine, Daniel S	57	0.16	Craske, Michelle G
3	78	0.11	Heimberg, Richard G	78	0.15	Rapee, Ronald M
4	78	0.15	Rapee, Ronald M	52	0.15	Hofmann, Stefan G
5	72	0.04	Buckner, Julia D	78	0.11	Heimberg, Richard G
6	68	0.11	Andersson, Gerhard	68	0.11	Andersson, Gerhard
7	57	0.16	Craske, Michelle G	53	0.07	Carlbring, Per
8	53	0.07	Carlbring, Per	49	0.07	Kendall, Philip C
9	52	0.15	Hofmann, Stefan G	82	0.06	Zvolensky, Michael J
10	49	0.07	Kendall, Philip C	19	0.06	De los reyes, Andres

#### 3.1.3 Analysis of countries and institutions

**Figure 5:**
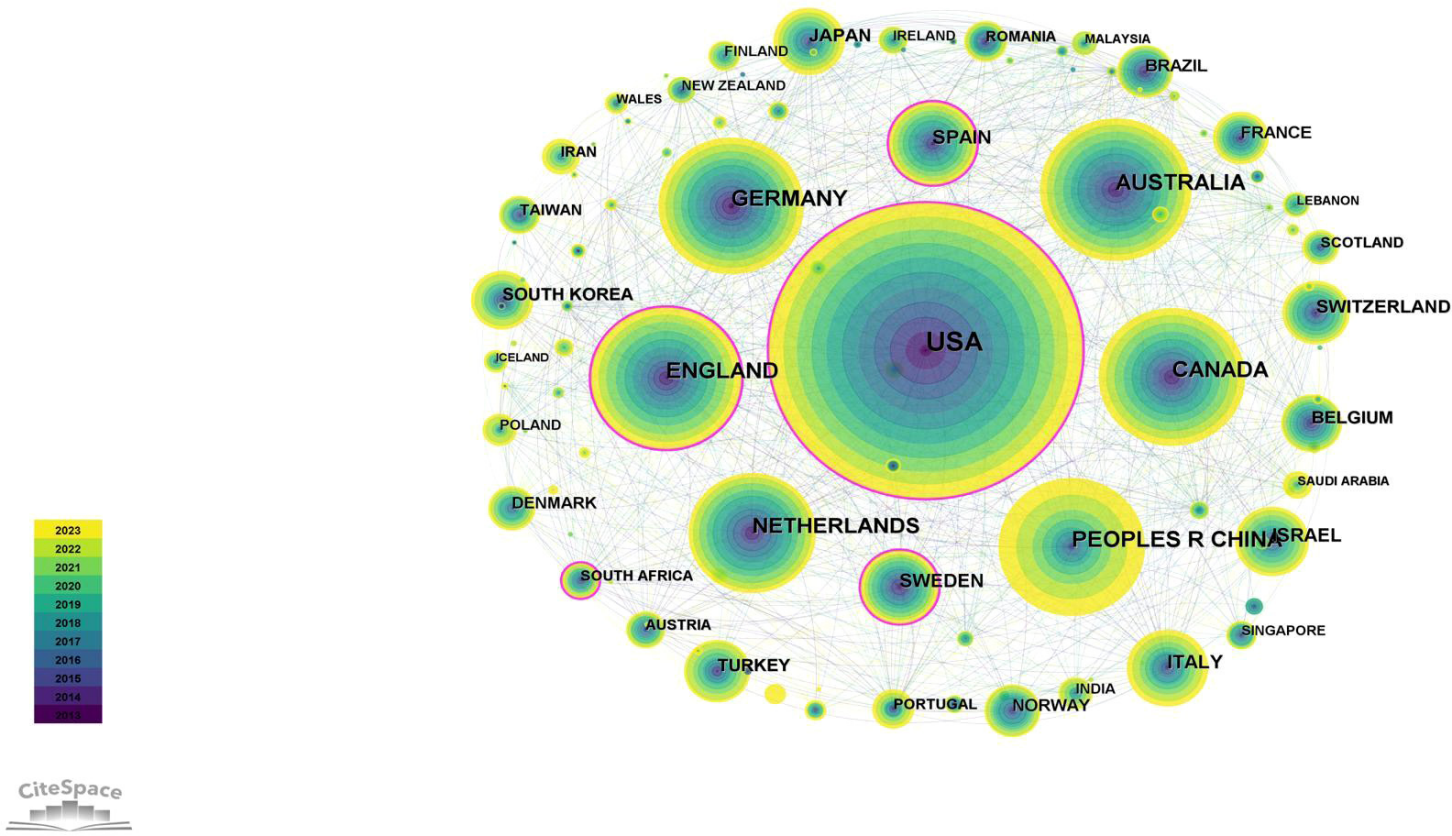
The collaboration network of countries related to SA.

**Figure 6:**
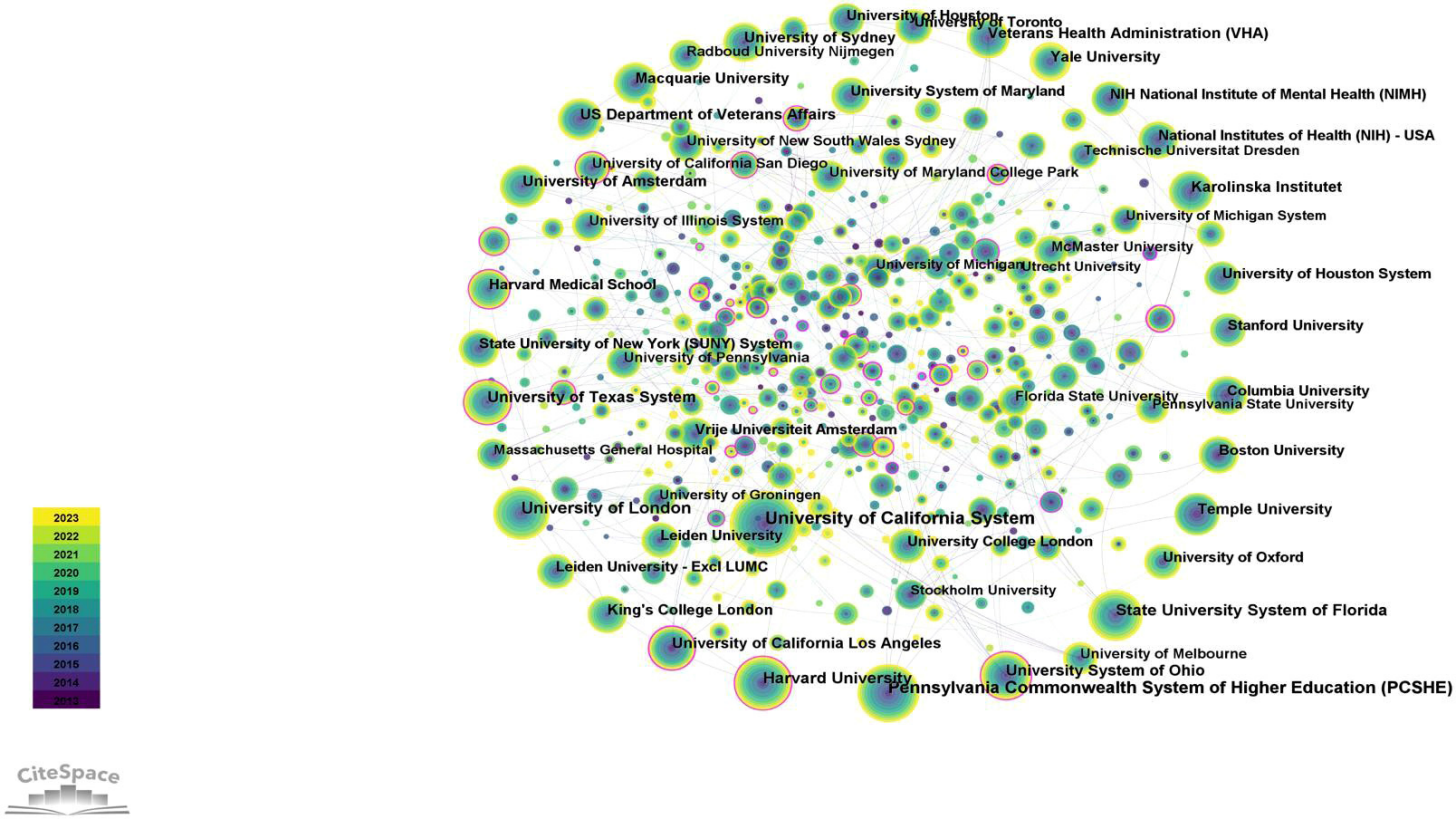
The collaboration network of institutions related to SA.

CiteSpace was used to analyze national and institutional cooperation networks. In collaborative networks obtained by CiteSpace, the size of nodes represents the number of publications published by a certain organization or country. The results indicated that the USA ranks first, with 3952 (39.76%) publications (**Table 2**), followed by Australia (987, 9.93%) and England (961, 9.67%). As shown in the national collaborations network of SA (**Figure 5**), the USA is the leading country working closely with other countries. In addition, although China ranks fourth with 914 articles, its centrality is only 0.02, which is lower than other countries. In terms of the number of institutional publications (**Figure 6**; **Table 3**), seven of the top 10 organizations/universities are from the USA, accounting for 21.04% of the total number of publications, which is enough to illustrate the prominent role of the USA in the field of SA research.

**Table 2:** The top 10 countries related to SA.

Rank	Publications	Centrality	Country	Percent
1	3952	0.11	USA	39.76%
2	987	0.06	Australia	9.93%
3	961	0.12	England	9.67%
4	914	0.02	Peoples R China	9.20%
5	904	0.05	Canada	9.09%
6	867	0.09	Germany	8.72%
7	681	0.07	Netherlands	6.85%
8	352	0.13	Spain	3.54%
9	311	0.08	Italy	3.13%
10	282	0.11	Sweden	2.84%

**Table 3:** The top 10 institutions related to SA.

Rank	Publications	Centrality	Institution	Country
1	474	0.02	University of California System	USA
2	365	0.02	Pennsylvania Commonwealth System of Higher Education	USA
3	320	0.01	University of London	UK
4	310	0.03	Harvard University	USA
5	282	0.01	State University System of Florida	USA
6	241	0.03	University System of Ohio	USA
7	216	0.02	University of Texas System	USA
8	209	0.01	University of Amsterdam	Netherlands
9	203	0.01	US Department of Veterans Affairs	USA
10	201	0.01	Karolinska Institutet	Sweden

#### 3.1.4 Analysis of journals

Journal analysis can help scholars to understand the disciplinary attributes of the research object, and then deepen their understanding of the disciplinary connotation. The 9940 articles analyzed in this study were published in 1157 journals. **Table 4** shows the top 10 journals in the field of SA. In terms of publication, the Journal of Frontiers in Psychology (317) ranks first, followed by the Journal of Affective Disorders (300) and the Journal of Anxiety Disorders (267). In terms of Country/region, four are based in the USA, two are based in England, two are based in Switzerland, and two are based in the Netherlands. In terms of impact factor, Journal of Affective Disorders (4.9) was the highest, among the top 10 journals. In general, the top 10 journals accounted for 20.36% of the total number of publications, but the overall impact factor of the journals was not high.

**Table 4:** The top 10 journals related to SA.

Rank	Publications	Journal	Country/region	IF (2023)	JCR
1	317	Frontiers in Psychology	Switzerland	2.6	Q2
2	300	Journal of Affective Disorders	Netherlands	4.9	Q1
3	267	Journal of Anxiety Disorders	USA	4.8	Q1
4	198	Plos One	USA	2.9	Q1
5	178	Psychiatry Research	Netherlands	4.2	Q1
6	176	Behavior Research and Therapy	England	4.2	Q1
7	172	Cognitive Therapy and Research	USA	2.8	Q2
8	156	Frontiers in Psychiatry	Switzerland	3.2	Q2
9	142	Journal of Behavior Therapy and Experimental Psychiatry	England	1.7	Q3
10	118	Behavior Therapy	USA	3.4	Q1

**Table 5** shows the top 10 co-cited journals in the field of SA. The most frequent co-citations is Behavior Research and Therapy (5728), followed by Journal of Anxiety Disorders (5021) and Clinical Psychology Review (4406). Most of the journals are still based in the USA. In terms of impact factor, Three of the top 10 journals have an impact factor >10, including Psychological Bulletin (17.3), American Journal of Psychiatry (15.1), and American Journal of Psychiatry (13.7).

**Table 5:** The top 10 co-cited journals related to SA.

Rank	Citation	Journal	Country	IF (2023)	JCR
1	5728	Behavior Research and Therapy	England	4.2	Q1
2	5021	Journal of Anxiety Disorders	USA	4.8	Q1
3	4406	Clinical Psychology Review	USA	13.7	Q1
4	4044	Depression and Anxiety	USA	4.7	Q1
5	3991	Archives of General Psychiatry	USA	/	/
6	3942	Psychological Medicine	USA	5.9	Q1
7	3787	Journal of Abnormal Psychology	USA	/	/
8	3782	American Journal of Psychiatry	USA	15.1	Q1
9	3712	Psychological Bulletin	USA	17.3	Q1
10	3592	Journal of Consulting and Clinical Psychology	USA	4.5	Q1

### 3.2 Analysis of keywords

#### 3.2.1 Co-occurrence analysis of keywords

**Figure 7:**
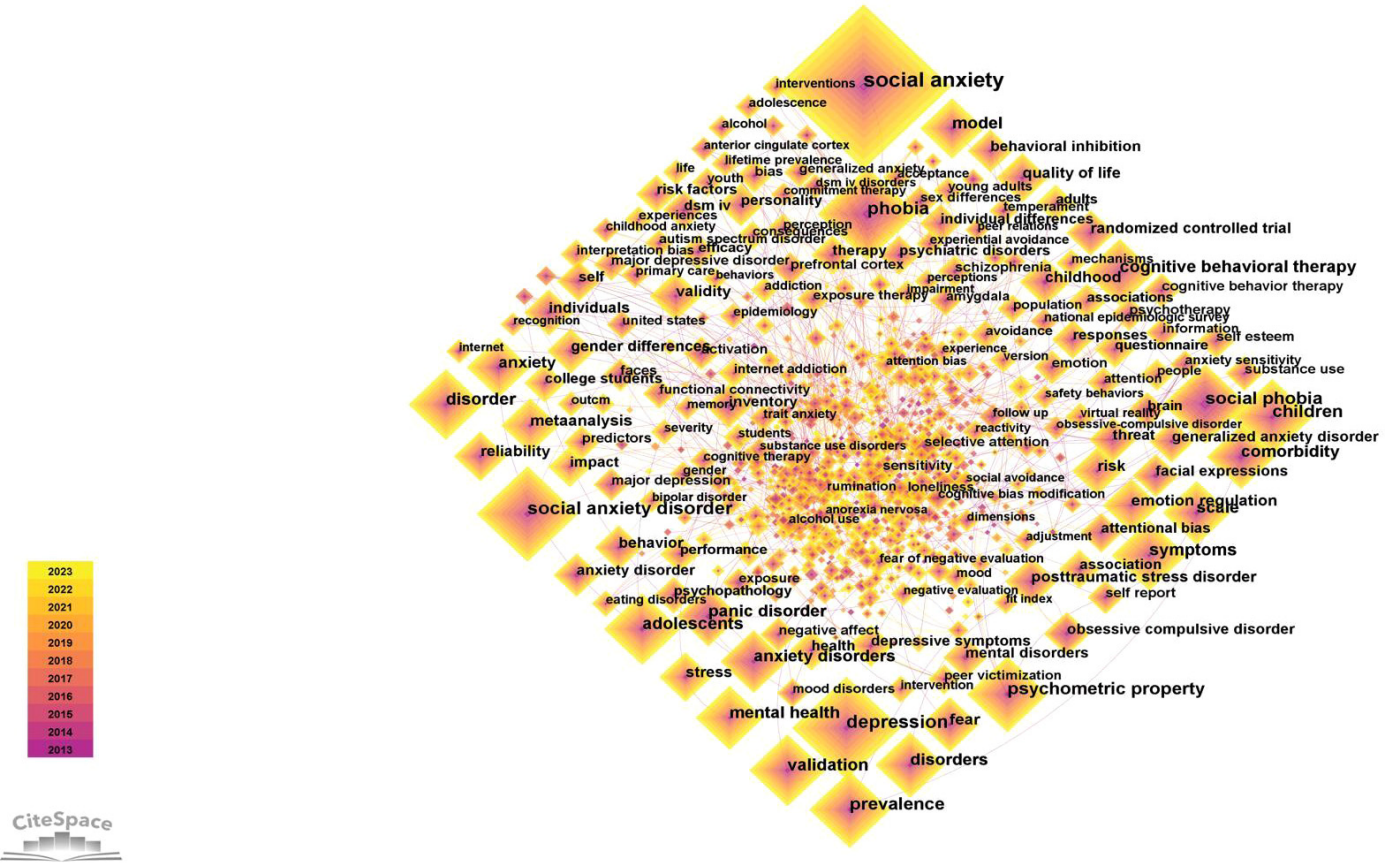
Keywords co-occurrence network.

Keywords are the essence of an article, and the analysis of keywords is helpful in understanding the research emphases and hot spots in this field. As shown in the **Figure 7**, the largest node in the co-occurrence map was SA, consonant with the theme of this study. This study selected the top 20 keywords of frequency and centrality (**Table 6**) to help understand the research hotspots in this field from 2013 to 2023. Through the classification of keywords, it can be found that keywords involve symptoms, psychological intervention, comorbidities, scientific research, and research objects. However, they do not exist in isolation but are closely related.

**Table 6:** Top 20 high-frequency and centrality keywords in the field of SA.

Rank	Frequency	Keyword	Centrality	Keyword
1	4382	social anxiety	0.02	social anxiety
2	1719	depression	0.02	social avoidance
3	1503	social anxiety disorder	0.02	phobia inventory
4	1451	phobia	0.02	attention deficit/hyperactivity disorder
5	1045	social phobia	0.02	decision making
6	977	psychometric property	0.02	prevention
7	977	children	0.02	patterns
8	963	prevalence	0.02	age of onset
9	857	adolescents	0.02	brief fear
10	855	validation	0.02	treatment response
11	847	symptoms	0.02	Asperger syndrome
12	808	disorder	0.02	child anxiety
13	751	cognitive behavioral therapy	0.02	deficits
14	725	disorders	0.02	depressive disorders
15	700	comorbidity	0.02	self focused attention
16	694	scale	0.02	childhood maltreatment
17	681	mental health	0.02	childhood trauma
18	667	anxiety disorders	0.01	social anxiety disorder
19	624	model	0.01	phobia
20	615	panic disorder	0.01	social phobia

#### 3.2.2 Cluster analysis of keywords

Keywords clustering map is used to analyze the similarity between keyword nodes in a field. Nodes with obvious relationships are clustered into a category based on data operations. The formation of this category contains a large number of keywords that can represent research hotspots in a certain field.

**Figure 8:**
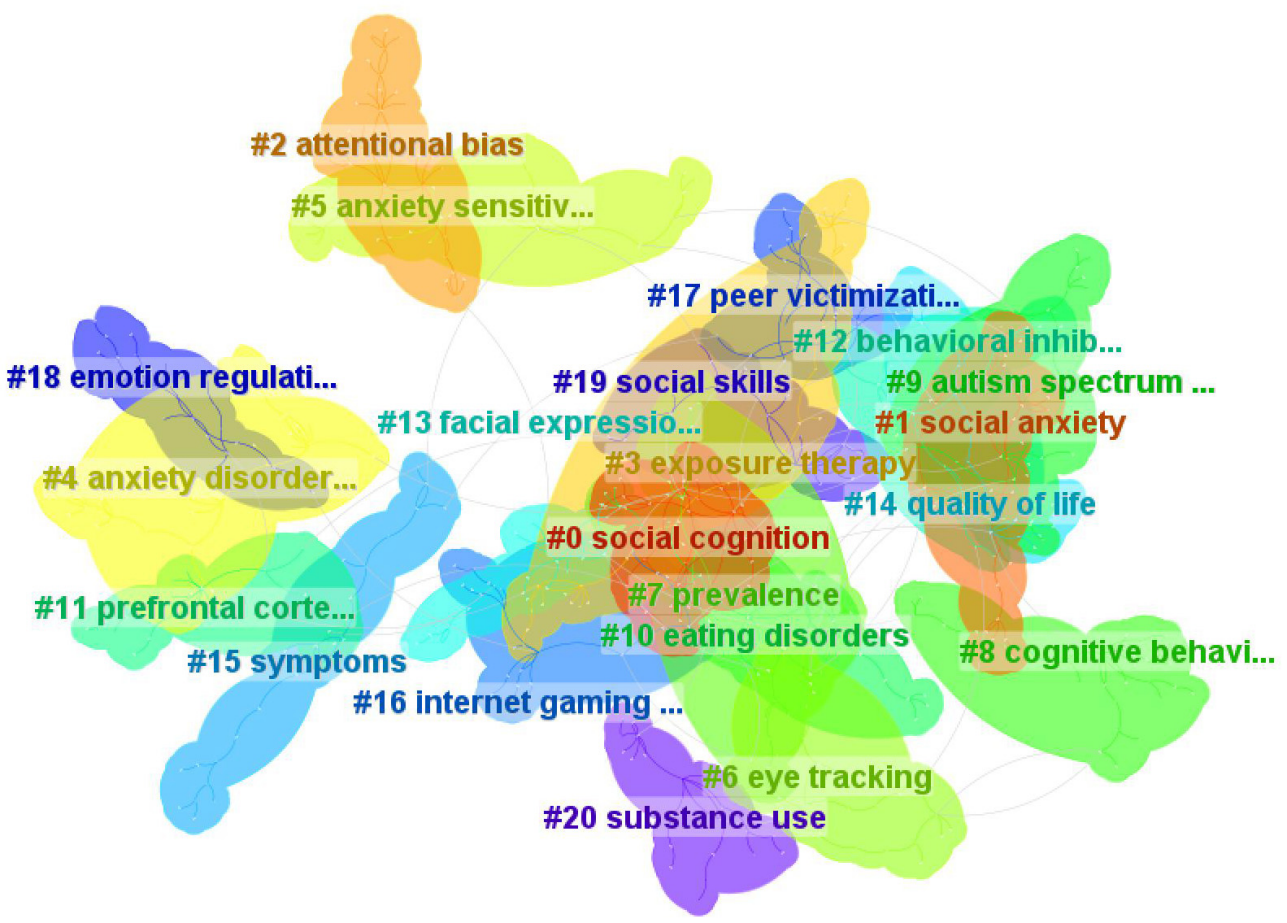
Keywords clustering map.

To further understand the themes of SA research over the past decade, further keyword clustering analysis was performed (**Figure 8**). The results show that there are 21 keyword clusters (**Table 7**). We can roughly classify the keyword clusters into the following themes: symptom(cognitive dysfunction #0, #2, #13, #18; behavioral symptom #12, #15), comorbid conditions (#4, #9, #10), intervention strategy(#3, #8, #19), negative effect(#14, #16, #20), risk factors(#5, #17), research techniques(#6), brain region(#11), and relatively independent clusters (#1, #7).

**Table 7:** Keywords each cluster information.

ClusterID	Size	Silhouette	Mean (Year)	Label (LLR)
0	35	0.874	2015	social cognition (94.55, 1.0E-4); borderline personality disorder (79.9, 1.0E-4); theory of mind (62.84, 1.0E-4); oxytocin (41.34, 1.0E-4); major depressive disorder (34.71, 1.0E-4)
1	35	0.976	2014	social anxiety (463.74, 1.0E-4); social anxiety disorder (191.56, 1.0E-4); fear of negative evaluation (129.35, 1.0E-4); fear of positive evaluation (94.48, 1.0E-4); measurement invariance (56.75, 1.0E-4)
2	33	0.973	2014	attentional bias (331.35, 1.0E-4); attention bias (232.22, 1.0E-4); interpretation bias (196.81, 1.0E-4); cognitive bias modification (188.11, 1.0E-4); attention bias modification (142.33, 1.0E-4)
3	32	1	2016	exposure therapy (250.58, 1.0E-4); virtual reality (217.51, 1.0E-4); randomized controlled trial (146.51, 1.0E-4); cognitive behavioral therapy (135.63, 1.0E-4); d-cycloserine (64.03, 1.0E-4)
4	31	0.963	2015	anxiety disorders (405.38, 1.0E-4); panic disorder (264.8, 1.0E-4); social anxiety (183.02, 1.0E-4); generalized anxiety disorder (153.06, 1.0E-4); posttraumatic stress disorder (104.09, 1.0E-4)
5	29	0.993	2016	anxiety sensitivity (186.51, 1.0E-4); positive affect (127.35, 1.0E-4); negative affect (82.31, 1.0E-4); autobiographical memory (52.05, 1.0E-4); working memory (24.69, 1.0E-4)
6	28	0.969	2017	eye tracking (68.94, 1.0E-4); internet addiction (65.16, 1.0E-4); repetitive negative thinking (49.37, 1.0E-4); negative evaluation (37.89, 1.0E-4); rumination (32.64, 1.0E-4)
7	28	1	2014	prevalence (145.13, 1.0E-4); comorbidity (123.97, 1.0E-4); anxiety disorder (85.98, 1.0E-4); mental disorders (80.31, 1.0E-4); substance use disorders (68.28, 1.0E-4)
8	27	1	2015	cognitive behavior therapy (92.51, 1.0E-4); post-event processing (55.08, 1.0E-4); systematic review (40.96, 1.0E-4); self help (35.55, 1.0E-4); meta -analysis (32.95, 1.0E-4)
9	26	0.955	2016	autism spectrum disorder (96.01, 1.0E-4); psychometric property (65.98, 1.0E-4); self report (44.94, 1.0E-4); scale (37.66, 1.0E-4); young people (34.02, 1.0E-4)
10	26	1	2016	eating disorders (283.08, 1.0E-4); anorexia nervosa (139.76, 1.0E-4); body image (115.57, 1.0E-4); body dysmorphic disorder (74.78, 1.0E-4); obsessive-compulsive disorder (64.86, 1.0E-4)
11	24	0.971	2016	prefrontal cortex (149.69, 1.0E-4); functional connectivity (142.63, 1.0E-4); amygdala (99.28, 1.0E-4); brain (72.97, 1.0E-4); fmri (69.53, 1.0E-4)
12	24	0.964	2016	behavioral inhibition (184.09, 1.0E-4); temperament (142.98, 1.0E-4); social avoidance (107.55, 1.0E-4); shyness (95.1, 1.0E-4); social withdrawal (69.69, 1.0E-4)
13	24	0.976	2014	facial expression (94.28, 1.0E-4); emotion (84.05, 1.0E-4); amygdala (77.24, 1.0E-4); sex differences (75.03, 1.0E-4); perception (64.87, 1.0E-4)
14	23	0.932	2015	quality of life (80.95, 1.0E-4); children (75.58, 1.0E-4); childhood (66.84, 1.0E-4); multiple informants (47.28, 1.0E-4); adolescents (41.9, 1.0E-4)
15	23	0.975	2015	symptoms (64.66, 1.0E-4); emotional disorders (35.9, 1.0E-4); reliability (31.36, 1.0E-4); population (27.37, 1.0E-4); impairment (22.55, 1.0E-4)
16	22	0.981	2016	internet gaming disorder (79.04, 1.0E-4); problematic internet use (62.92, 1.0E-4); internet addiction (61.21, 1.0E-4); loneliness (58.39, 1.0E-4); responses (52.48, 1.0E-4)
17	22	0.919	2015	peer victimization (139.4, 1.0E-4); victimization (59.25, 1.0E-4); bullying (49.3, 1.0E-4); follow up (43.55, 1.0E-4); experiences (37.73, 1.0E-4)
18	22	0.955	2015	emotion regulation (427.09, 1.0E-4); cognitive reappraisal (166.7, 1.0E-4); expressive suppression (119.25, 1.0E-4); reappraisal (76.77, 1.0E-4); individual differences (34.49, 1.0E-4)
19	22	0.902	2014	social skills (56.6, 1.0E-4); young adults (54.49, 1.0E-4); autism (36.05, 1.0E-4); autism spectrum disorders (29.36, 1.0E-4); social isolation (27.57, 1.0E-4)
20	21	0.982	2014	substance use (173.95, 1.0E-4); alcohol use (154.2, 1.0E-4); cannabis (131.5, 1.0E-4); alcohol (110.08, 1.0E-4); marijuana (98.36, 1.0E-4)

#### 3.2.3 Burst analysis of keywords

Burst keyword indicates that the keyword has been in high focus for a certain period of time. By plotting these keywords based on their burst strength and year, a burst map is created. The map reveals ongoing hot topics and highlights the most intense areas of research, helping researchers track trends and frontiers.

**Figure 9:**
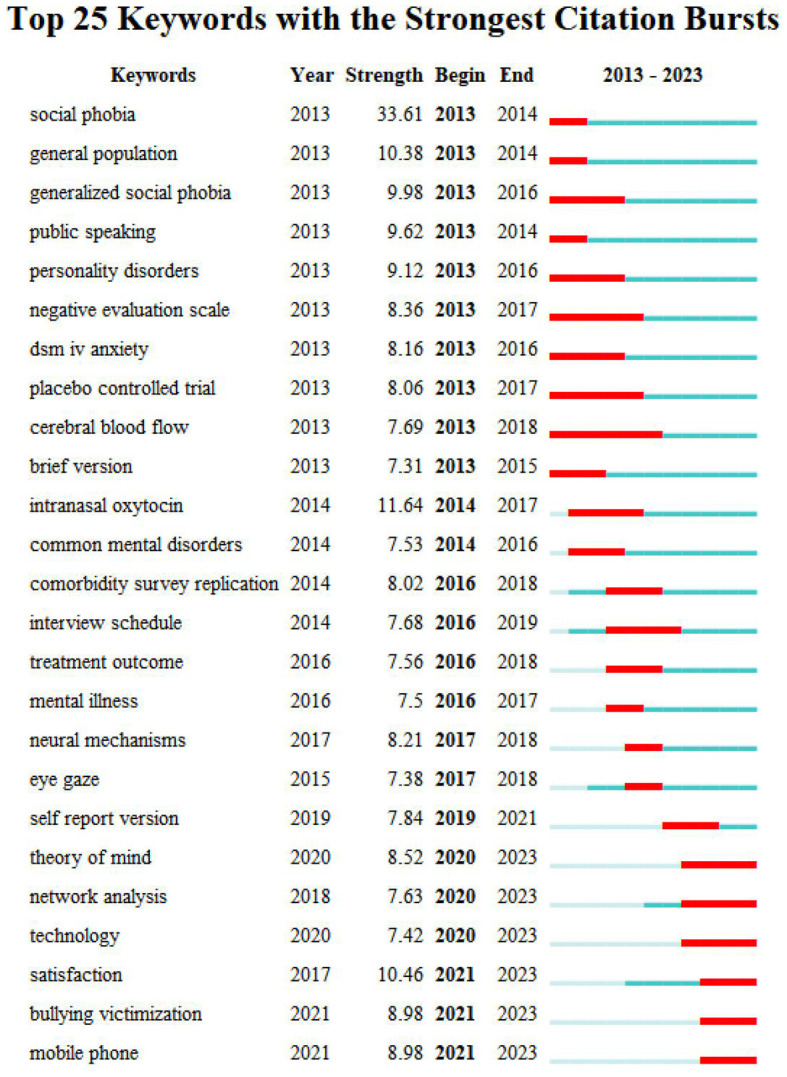
Top 25 keywords with strongest citation bursts.

The burst keywords can be obtained by using the burstness function of CiteSpace. Through analysis, a total of 25 burst keywords were obtained, and the specific results can be seen in **Figure 9**. Taking 2023 as the cutoff time for burst, we found that the keywords that have proliferated in recent years are: “theory of mind”, “network analysis”, “technology”, “satisfaction”, “bullying victimization”, and “mobile phone”. These topics may be at the forefront of future research in the field of SA.

### 3.3 Analysis of co-cited references

**Figure 10:**
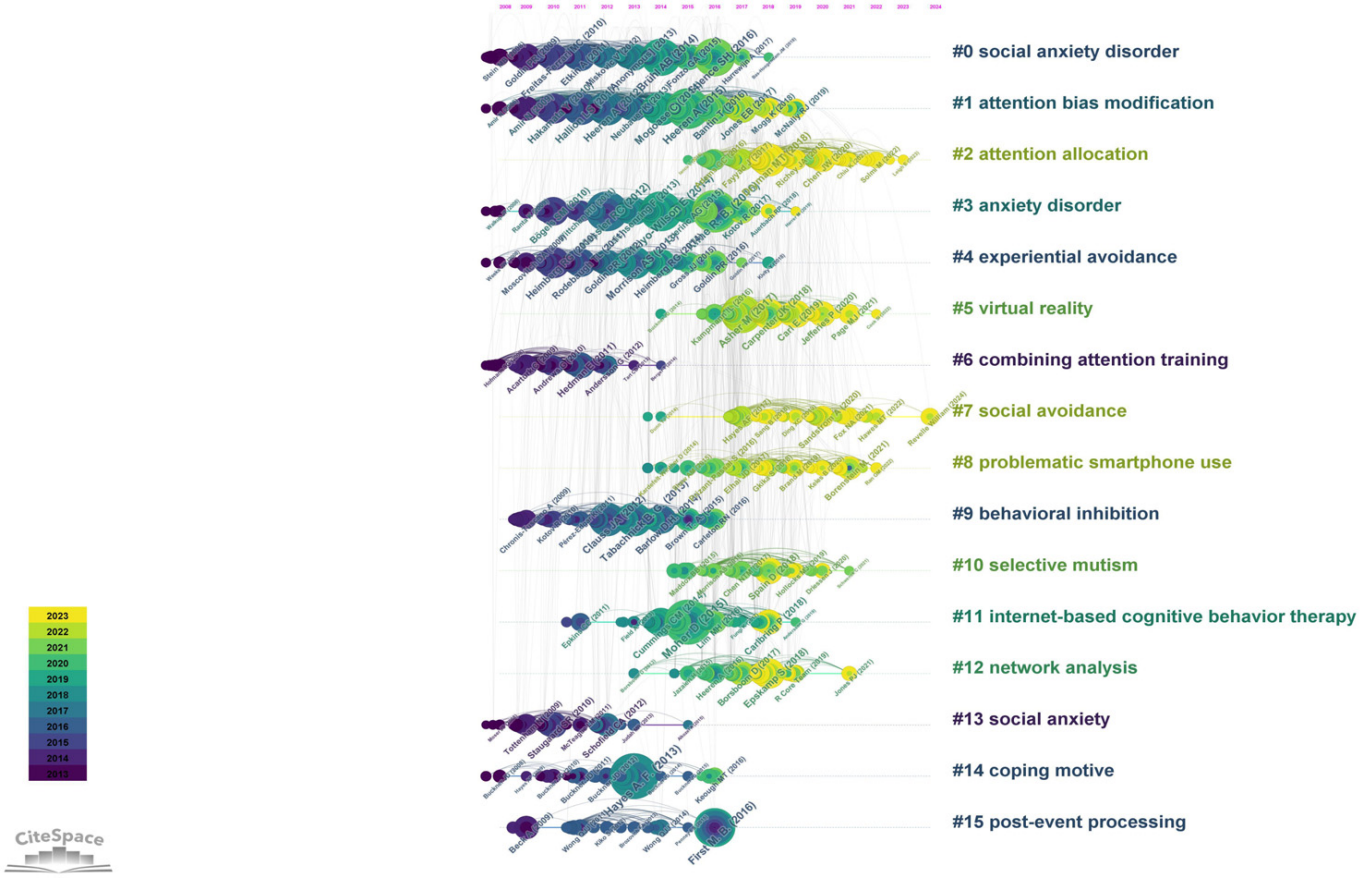
Timeline view of co-cited references related to SA.

Every article cites a number of references. These references are represented as nodes in a co-citation network. The connectivity between the nodes of a reference indicates how often they are cited by the same article. The network formed in this way captures the research focus of the basic science community (20). **Figure 10** shows a timeline view of the cited reference in SA research. In **Figure 10**, each nodes represents each cited reference, while the size of the nodes indicates how often the reference was cited. CiteSpace divides the co-citation network into multiple clusters of co-cited reference such that the literature within the same cluster is closely linked and arranged in descending order of the number of literature contained in the cluster. In this paper, the five most important clusters will be analyzed (**Table 8**).

**Table 8:** Major clusters of co-cited references.

ClusterID	Size	Silhouette	mean(Year)	Label (LLR)
0	147	0.909	2012	social anxiety disorder (2942.16, 1.0E-4); generalized anxiety disorder (2677.77, 1.0E-4); functional connectivity (2162.61, 1.0E-4); neural correlate (1966.48, 1.0E-4); cortical thickness (1732.06, 1.0E-4)
1	136	0.869	2013	attention bias modification (10223.89, 1.0E-4); attentional bias modification (6138.64, 1.0E-4); attentional bia (4330.13, 1.0E-4); cognitive bias modification (2856.98, 1.0E-4); attention bias modification training (2352.02, 1.0E-4)
2	119	0.729	2019	attention allocation (1978.5, 1.0E-4); negative interpretation (1859.93, 1.0E-4); interpretation bia (1605.71, 1.0E-4); social anxiety (1505.02, 1.0E-4); interpretation bias task (1258.92, 1.0E-4)
3	113	0.815	2013	anxiety disorder (5632.3, 1.0E-4); treatment outcome (3170.66, 1.0E-4); childhood anxiety disorder (2773.16, 1.0E-4); fear extinction (1866.66, 1.0E-4); error-related brain activity (1764.53, 1.0E-4)
4	112	0.87	2012	experiential avoidance (2597.76, 1.0E-4); cognitive reappraisal (1739.63, 1.0E-4); emotion regulation (1687.37, 1.0E-4); cardiovascular responses (1359.52, 1.0E-4); positive v (1351.6, 1.0E-4)

**Figure 11:**
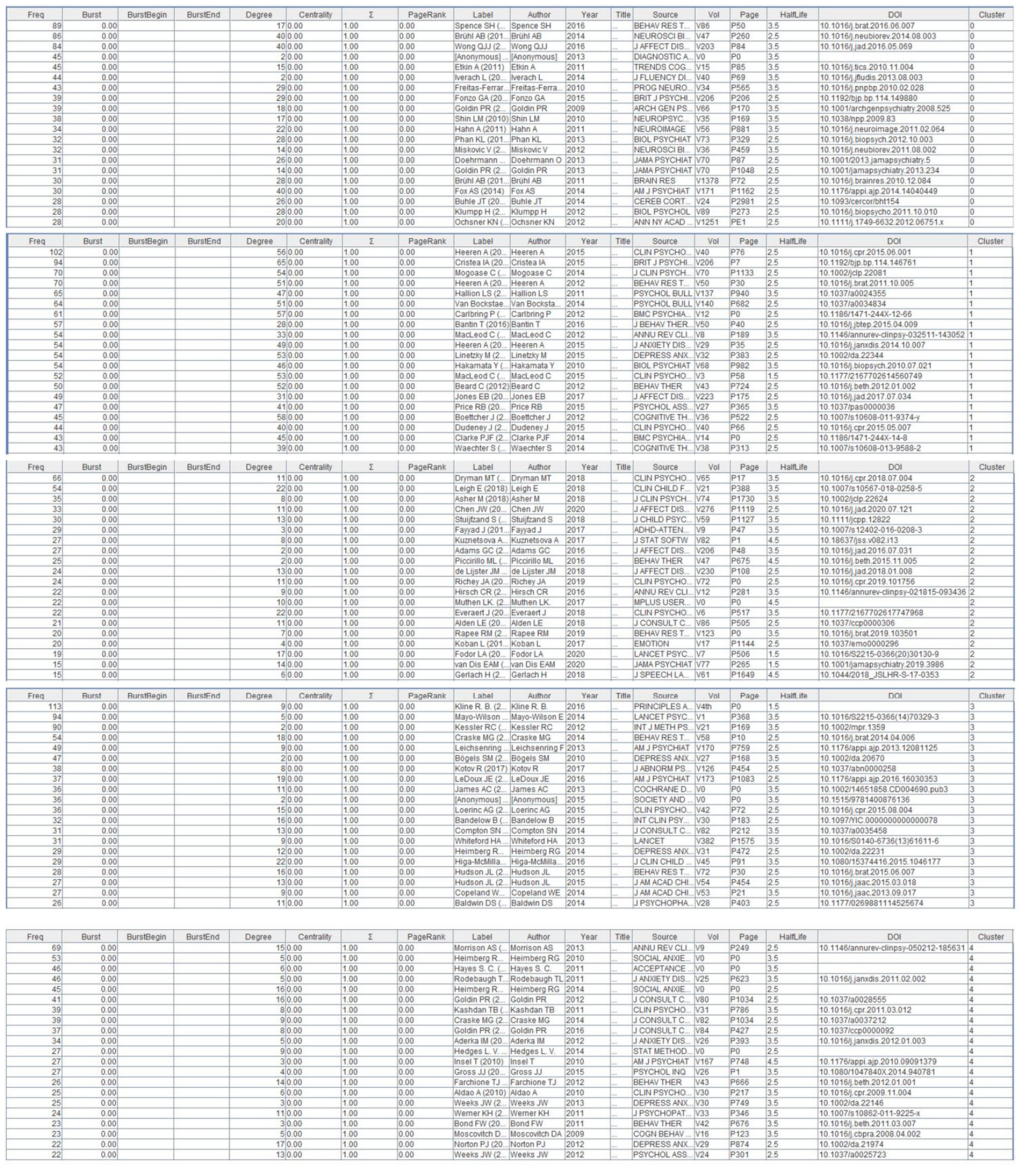
Top 20 cited references within each co-citation cluster.

Cluster #0 social anxiety disorder has the largest number of nodes and contains 147 cited references with an average citation year of 2012, it is the most dominant cluster. **Figure 11** lists the top 20 cited references for this cluster. As can be seen from the timeline, one of the largest nodes of this cluster appeared in 2016, and this article is about the SA etiology model proposed by Spence et al. in 2016. The model emphasizes the complex pathways of SA development and the interplay between environmental and intrinsic factors, pointing to the need for further longitudinal research in the future (13). Another equally landmark piece of literature is the 2014 article by Brühl et al. They proposed a new model of the neurobiology of SA that confirmed the hyperactivation of fear circuits (amygdala, insula, anterior cingulate gyrus, and prefrontal cortex) in SA (21). These studies can help researchers better understand the pathogenesis of SA and provide guidance for its prevention and treatment. Analyzing the citing articles of #0 can also help us to better understand which area of SA the references in this cluster contribute more to the research. Reading and combing through the top 20 citing papers revealed that most articles covered neuroimaging studies of SA, which also coincided with the main core keywords of the cluster (**Table 9**). More specifically, studies covering neurobiology (2, 5, 7, 16), cognitive mechanisms (1, 2, 6, 12, 13, 15, 18, 20), theoretical modeling (4, 19), and intervention strategies (9, 20). In summary, reading of this clustered citation literature will greatly contribute to our deeper understanding of the neural mechanisms of SA.

**Table 9:** Citing articles to cluster #0.

Rank	Coverage	Bibliography
1	29	Mogg, K (2016.0) Anxiety and attention to threat: cognitive mechanisms and treatment with attention bias modification.
2	28	Bas-hoogendam, JM (2016.0) Neurobiological candidate endophenotypes of social anxiety disorder.
3	26	Bruehl, AB (2014.0) Neuroimaging in social anxiety disorder-a meta-analytic review resulting in a new neurofunctional model.
4	25	Wong, QJJ (2016.0) The etiology and maintenance of social anxiety disorder: a synthesis of complimentary theoretical models and formulation of a new integrated model.
5	19	Shackman, AJ (2016.0) Dispositional negativity: an integrative psychological and neurobiological perspective.
6	18	Schulz, C (2013.0) Automatic neural processing of disorder-related stimuli in social anxiety disorder: faces and more.
7	15	Bas-hoogendam, JM (2018.0) Subcortical brain volumes, cortical thickness and cortical surface area in families genetically enriched for social anxiety disorder - a multiplex multigenerational neuroimaging study.
8	14	Duval, ER (2015.0) Neural circuits in anxiety and stress disorders: a focused review.
9	14	Richter, J (2017.0) Bridging the gaps between basic science and cognitive-behavioral treatments for anxiety disorders in routine care.
10	14	Blackford, JU (2014.0) Amygdala-cingulate intrinsic connectivity is associated with degree of social inhibition.
11	14	Bruehl, AB (2014.0) Increased cortical thickness in a frontoparietal network in social anxiety disorder.
12	14	Taylor, CT (2014.0) Neural correlates of a computerized attention modification program in anxious subjects.
13	13	Britton, JC (2015.0) Neural changes with attention bias modification for anxiety: a randomized trial.
14	12	Bas-hoogendam, JM (2017.0) Voxel-based morphometry multi-center mega-analysis of brain structure in social anxiety disorder.
15	12	Cremers, HR (2016.0) Social anxiety disorder: a critical overview of neurocognitive research.
16	11	Bas-hoogendam, JM (2018.0) The leiden family lab study on social anxiety disorder: a multiplex, multigenerational family study on neurocognitive endophenotypes.
17	11	Wang, X (2018.0) Gray matter structural alterations in social anxiety disorder: a voxel-based meta-analysis.
18	11	Bruehl, AB (2013.0) General emotion processing in social anxiety disorder: neural issues of cognitive control.
19	11	Spence, SH (2016.0) The etiology of social anxiety disorder: an evidence-based model.
20	11	Klumpp, H (2017.0) Predicting cognitive behavioral therapy response in social anxiety disorder with anterior cingulate cortex and amygdala during emotion regulation.

Cluster #1 Attention Bias Modification contains 136 citations with an average citation year of 2013. Heeren is a key author in this cluster. Two of the top five cited references are from Heeren (**Figure 11**). The landmark article in this cluster is the article published by Heeren et al. in 2015. In this study, researchers provided a comprehensive assessment of the effect of Attention bias modification (ABM) in the treatment of SA (22). Additionally, another study further confirmed the effectiveness of Attention Training in reducing SA symptoms and more able to shift attention away from threatening stimuli reducing the focus on potential threats in social situations. The citing literature for this cluster focuses on the use and effects of ABM in SA (**Table 10**). A study by Mogg et al. (2017) explored the effects of multiple sessions of ABM training on reducing attentional bias in individuals with high anxiety (23). Macleod (2016) emphasized the importance of clarity in bias processes and assessment procedures to deepen the understanding of the mechanism of action of ABM (24). Second, several studies have revealed the neurobiological mechanisms of attentional bias, in particular the role of negative emotional tendencies, threat perception, and age. These findings help to explain the influence of individual differences on the effects of ABM (25). In addition, a study by Boettcher et al. (2014) explored the potential effects of combining ABM with cognitive behavioral therapy (CBT) in the context of online self-help therapy (26). However, future research needs to further explore the short- and long-term effects of ABM and its applicability in different populations to ensure the breadth and effectiveness of its clinical application.

**Table 10:** Citing articles to cluster #1.

Rank	Coverage	Bibliography
1	28	Heeren, A (2015.0) Attention bias modification for social anxiety: a systematic review and meta-analysis.
2	27	Macleod, C (2016.0) Anxiety-linked attentional bias and its modification: illustrating the importance of distinguishing processes and procedures in experimental psychopathology research.
3	27	Mogg, K (2017.0) Attention bias modification (abm): review of effects of multisession abm training on anxiety and threat-related attention in high-anxious individuals.
4	26	Boettcher, J (2013.0) Internet-based interventions for social anxiety disorder - an overview.
5	26	Mogg, K (2016.0) Anxiety and attention to threat: cognitive mechanisms and treatment with attention bias modification.
6	25	Shackman, AJ (2016.0) The neurobiology of dispositional negativity and attentional biases to threat: implications for understanding anxiety disorders in adults and youth.
7	21	Fu, X (2019.0) Threat-related attention bias in socioemotional development: a critical review and methodological considerations.
8	21	Abend, R (2019.0) Age moderates link between training effects and treatment response to attention bias modification treatment for social anxiety disorder.
9	21	Boettcher, J (2013.0) Combining attention training with cognitive-behavior therapy in internet-based self-help for social anxiety: study protocol for a randomized controlled trial.
10	21	Liu, H (2017.0) Effects of cognitive bias modification on social anxiety: a meta-analysis.
11	21	Price, RB (2016.0) Pooled patient-level meta-analysis of children and adults completing a computer-based anxiety intervention targeting attentional bias.
12	21	Mogoase, C (2014.0) Clinical efficacy of attentional bias modification procedures: an updated meta-analysis.
13	20	Dennis-tiwary, TA (2016.0) For whom the bell tolls: neurocognitive individual differences in the acute stress-reduction effects of an attention bias modification game for anxiety.
14	20	Mcnally, RJ (2019.0) Attentional bias for threat: crisis or opportunity?
15	19	De, voogd EL (2017.0) Online visual search attentional bias modification for adolescents with heightened anxiety and depressive symptoms: a randomized controlled trial.
16	18	Yao, N (2015.0) Does attention redirection contribute to the effectiveness of attention bias modification on social anxiety?
17	18	Carleton, RN (2015.0) A randomized controlled trial of attention modification for social anxiety disorder.
18	18	Boettcher, J (2014.0) Combining attention training with internet-based cognitive-behavioral self-help for social anxiety: a randomized controlled trial.
19	18	de, voogd EL (2016.0) Online attentional bias modification training targeting anxiety and depression in unselected adolescents: short- and long-term effects of a randomized controlled trial.
20	17	Linetzky, M (2015.0) Quantitative evaluation of the clinical efficacy of attention bias modification treatment for anxiety disorders.

Cluster #2 attention allocation is a relatively new cluster with an average citation year of 2019 and contains 136 cited references. Most of the high-impact cited references are in 2018 (**Figure 11**). Dryman et al.’s publication is a landmark contributing study in this cluster that confirms that SA is associated with underutilized cognitive reappraisal and overuse of emotional inhibition (27). Leigh et al.’s study focuses on understanding the contribution of cognitive modeling to socially anxious adolescents (28). The information citing articles (**Table 11**) on therapeutic approaches (3, 4, 20), emotion regulation (5, 13, 16), academic achievement (10), and neural mechanisms (6, 14) can also deepen our understanding of the mechanisms underlying attentional allocation in socially anxious individuals.

**Table 11:** Citing articles to cluster #2.

Rank	Coverage	Bibliography
1	14	Rapee, RM (2023.0) Anxiety disorders in children and adolescents: a summary and overview of the literature.
2	13	Olson, CM (2021.0) Familial factors in the development of social anxiety disorder.
3	13	Zainal, NH (2021.0) Pilot randomized trial of self-guided virtual reality exposure therapy for social anxiety disorder.
4	10	Luoma, J (2021.0) Mdma-assisted therapy as a means to alter affective, cognitive, behavioral, and neurological systems underlying social dysfunction in social anxiety disorder.
5	8	Akkus, K (2022.0) Exploring the relationship between interpersonal emotion regulation and social anxiety symptoms: the mediating role of negative mood regulation expectancies.
6	7	Zhang, Y (2023.0) From fears of evaluation to social anxiety: the longitudinal relationships and neural basis in healthy young adults.
7	7	Leigh, E (2023.0) Cognitive and behavioral processes in adolescents with social anxiety disorder.
8	7	Chiu, K (2021.0) Prospective associations between peer functioning and social anxiety in adolescents: a systematic review and meta-analysis.
9	7	Xia, T (2023.0) A study of the relationship between social anxiety and mask-wearing intention among college students in the post-covid-19 era: mediating effects of self-identity, impression management, and avoidance.
10	6	Leigh, E (2021.0) Is concentration an indirect link between social anxiety and educational achievement in adolescents?
11	6	Rogers, J (2022.0) A single-session online cognitive bias modification of interpretations modified for adults with anxiety and depressive symptoms.
12	6	Borghese, F (2022.0) Targeted memory reactivation during rem sleep in patients with social anxiety disorder.
13	6	Polack, RG (2023.0) Social interpretation inflexibility moderates emotional reactions to social situations in children and adolescents.
14	6	Zhang, X (2023.0) Large-scale brain functional network abnormalities in social anxiety disorder.
15	6	Pitcho-prelorentzos, S (2020.0) Predictors of social anxiety among online dating users.
16	6	Rozen, N (2023.0) Emotions in social anxiety disorder: a review.
17	6	Schaeuffele, C (2022.0) Transdiagnostic processes as mediators of change in an internet-delivered intervention based on the unified protocol.
18	6	Leung, CJ (2022.0) The combined cognitive bias hypothesis in anxiety: a systematic review and meta-analysis.
19	6	Fadardi, JS (2023.0) Scary in the eye of the beholder: attentional bias and attentional retraining for social anxiety.
20	6	Winter, HR (2023.0) Remote cognitive behavior therapy for social anxiety disorder: a meta-analysis.

The average citation year for cluster #3 anxiety disorder is 2013 and contains 113 citations. The most important cited reference for this cluster is Kline’s principles and practice of structural equation modeling(SEM) (**Figure 11**). This research methodology is widely used to explore the underlying dependent variables and complex psychological structures behind SA (29). For example, the study of SA involves multiple latent variables, such as mental toughness, self-esteem, social support, etc. SEM provides a framework that enables researchers to simultaneously examine the interactions and causal relationships among these variables, which not only deepens our understanding of the complex underlying mechanisms of SA but also facilitates the exploration of its pathways of intervention. While the literature in cluster #0 emphasized more on neuroimaging studies of SA, the content of the citing literature covered in #3 focused more on the treatment (**Table 12**), including Internet-delivered psychological treatment (1), treated in community clinic treatment (7), cognitive behavior therapy (10, 11, 15, 17, 20), monotherapy (14), transdiagnostic bibliotherapy program (19), and other therapies for SA and their predictors. Other factors that have an impact on the effectiveness of therapies such as negative emotions) (2, 5) and co-morbidities (6) are also addressed.

**Table 12:** Citing articles to cluster #3.

Rank	Coverage	Bibliography
1	22	Arnberg, FK (2014.0) Internet-delivered psychological treatments for mood and anxiety disorders: a systematic review of their efficacy, safety, and cost-effectiveness.
2	15	Shackman, AJ (2016.0) Dispositional negativity: an integrative psychological and neurobiological perspective.
3	13	Craske, MG (2017.0) Anxiety disorders.
4	12	Pittig, A (2018.0) The role of associative fear and avoidance learning in anxiety disorders: gaps and directions for future research.
5	12	Shackman, AJ (2016.0) The neurobiology of dispositional negativity and attentional biases to threat: implications for understanding anxiety disorders in adults and youth.
6	11	Walczak, M (2018.0) Does comorbidity predict poorer treatment outcome in pediatric anxiety disorders? an updated 10-year review.
7	10	Kodal, A (2018.0) Predictors of long-term outcome of cbt for youth with anxiety disorders treated in community clinics.
8	10	Morrison, AS (2016.0) Anxiety trajectories in response to a speech task in social anxiety disorder: evidence from a randomized controlled trial of cbt.
9	10	Andrews, G (2018.0) Royal Australian and New Zealand college of psychiatrists clinical practice guidelines for the treatment of panic disorder, social anxiety disorder and generalized anxiety disorder.
10	10	Richter, J (2017.0) Bridging the gaps between basic science and cognitive-behavioral treatments for anxiety disorders in routine care.
11	9	Cuijpers, P (2016.0) How effective are cognitive behavior therapies for major depression and anxiety disorders? a meta-analytic update of the evidence.
12	8	Kennedy, SM (2018.0) Predictors of treatment outcome for the unified protocol for transdiagnostic treatment of emotional disorders in children (up-c).
13	8	Steinert, C (2017.0) The effects of waiting for treatment: a meta-analysis of waitlist control groups in randomized controlled trials for social anxiety disorder.
14	8	Taylor, JH (2018.0) Monotherapy insufficient in severe anxiety? predictors and moderators in the child/adolescent anxiety multimodal study.
15	8	Kodal, A (2018.0) Long-term effectiveness of cognitive behavioral therapy for youth with anxiety disorders.
16	7	Leigh, E (2018.0) Understanding social anxiety disorder in adolescents and improving treatment outcomes: applying the cognitive model of Clark and wells (1995).
17	7	Stangier, U (2016.0) New developments in cognitive-behavioral therapy for social anxiety disorder.
18	7	Pelissolo, A (2019.0) Therapeutic strategies for social anxiety disorder: where are we now?
19	6	Wootton, BM (2018.0) An evaluation of the effectiveness of a transdiagnostic bibliotherapy program for anxiety and related disorders: results from two studies using a benchmarking approach.
20	6	Waters, AM (2018.0) Predicting outcomes for anxious children receiving group cognitive-behavioral therapy: does the type of anxiety diagnosis make a difference?

Cluster #4 experiential avoidance contains 112 citations with an average citation year of 2012. The study by Morrison et al. (2013) is an important article within this cluster, presenting an explanatory model of SA that includes aspects of information processing bias, emotion regulation, and safety behaviors (30) (**Figure 11**). Also of interest for this clustering is an article by Heimberg et al. (2010) entitled A cognitive behavioral model of social anxiety disorder: update and extension (31). According to the model, SA is partly caused by the individual’s overperception of social expectations and goals, which produces SA, further increases preoccupation with the self, and triggers negative cognitions, as a result, they negatively anticipate social situations and engage in avoidance and safety behaviors (experiential avoidance is itself a safety behavior). After the social event, they ruminate, reviewing their performance in the event and making negative evaluations of themselves, which in turn exacerbates SA. This creates a vicious cycle that leads to the maintenance and further worsening of SA. Kashdan et al. further revealed the effects of experiential avoidance on SA in both laboratory and naturalistic settings through two experiments and showed that the effect of experiential avoidance depended on the level of social threat and opportunity (32) (**Table 13**).

**Table 13:** Citing articles to cluster #4.

Rank	Coverage	Bibliography
1	29	Katzman, MA (2014.0) Canadian clinical practice guidelines for the management of anxiety, posttraumatic stress and obsessive-compulsive disorders.
2	19	Gilboa-Schectman, E (2013.0) More than a face: a unified theoretical perspective on nonverbal social cue processing in social anxiety.
3	17	Wong, QJJ (2016.0) The aetiology and maintenance of social anxiety disorder: a synthesis of complimentary theoretical models and formulation of a new integrated model.
4	14	Newby, JM (2014.0) Effectiveness of transdiagnostic internet cognitive behavioral treatment for mixed anxiety and depression in primary care.
5	12	Shackman, AJ (2016.0) Dispositional negativity: an integrative psychological and neurobiological perspective.
6	11	Wong, QJJ (2016.0) A review of scales to measure social anxiety disorder in clinical and epidemiological studies.
7	11	Pearl, SB (2017.0) Transdiagnostic versus diagnosis specific cognitive behavioral therapies for anxiety: a meta-analysis.
8	11	Moscovitch, DA (2013.0) Within the mind’s eye: negative mental imagery activates different emotion regulation strategies in high versus low socially anxious individuals.
9	10	Olthuis, JV (2014.0) Telephone-delivered cognitive behavioral therapy for high anxiety sensitivity: a randomized controlled trial.
10	10	Dryman, MT (2018.0) Emotion regulation in social anxiety and depression: a systematic review of expressive suppression and cognitive reappraisal.
11	10	Jazaieri, H (2015.0) The role of emotion and emotion regulation in social anxiety disorder.
12	9	Dryman, MT (2015.0) Examining the relationships among social anxiety, fears of evaluation, and interpretation bias.
13	9	Boettcher, J (2014.0) Internet-based mindfulness treatment for anxiety disorders: a randomized controlled trial.
14	9	Moscovitch, DA (2013.0) Self-portrayal concerns and their relation to safety behaviors and negative affect in social anxiety disorder.
15	9	Sapach, MJNT (2015.0) Cognitive constructs and social anxiety disorder: beyond fearing negative evaluation.
16	8	Kashdan, TB (2014.0) A contextual approach to experiential avoidance and social anxiety: evidence from an experimental interaction and daily interactions of people with social anxiety disorder.
17	8	Weeks, JW (2015.0) Psychometric evaluation of the concerns of social reprisal scale: further explicating the roots of fear of positive evaluation.
18	8	Epkins, CC (2016.0) Experiential avoidance and anxiety sensitivity: independent and specific associations with children’s depression, anxiety, and social anxiety symptoms.
19	8	Piccirillo, ML (2016.0) Safety behaviors in adults with social anxiety: review and future directions.
20	8	Norton, AR (2016.0) The efficacy of imagery rescripting compared to cognitive restructuring for social anxiety disorder.

**Figure 12:**
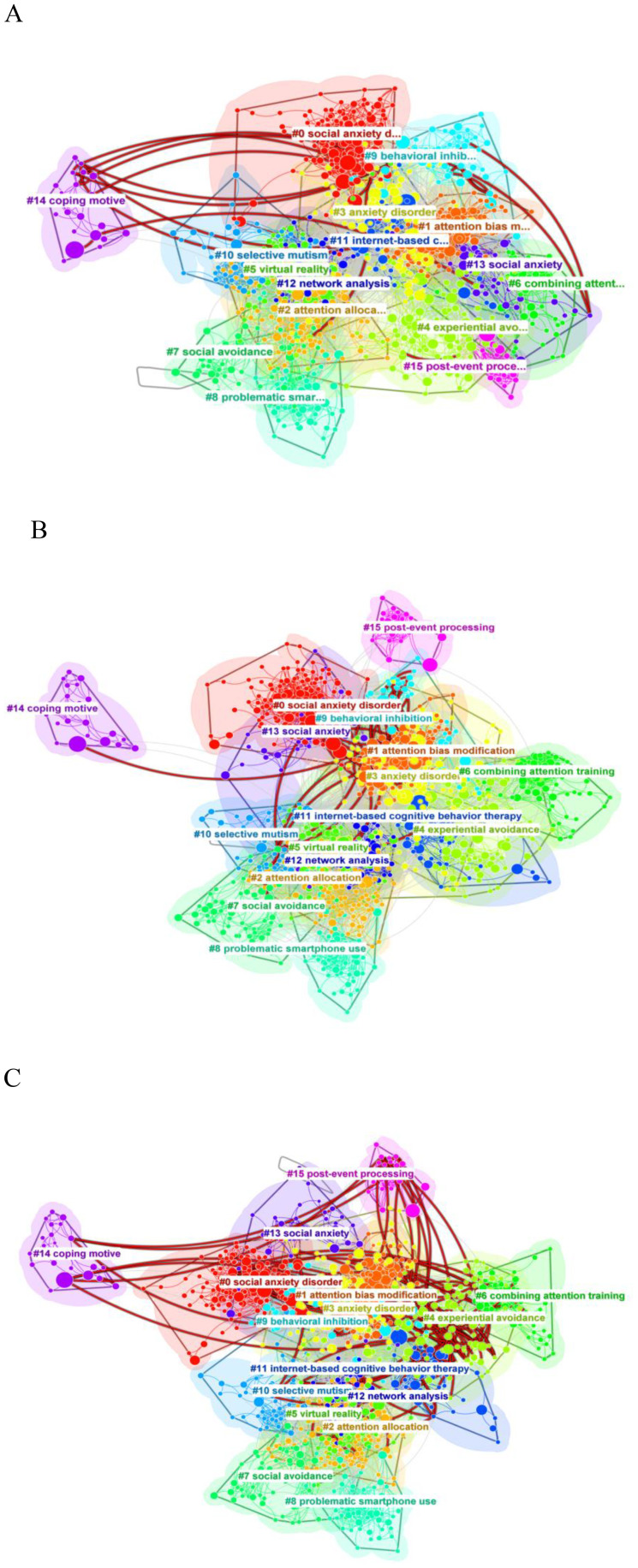
Authors ‘s citation trajectories. **(A)** Zvolensky, Michael J ‘s citation trajectories. **(B)** Pine, Daniel S ‘s citation trajectories. **(C)** Heimberg, Richard G ‘s citation trajectories.

In addition, the layer view function of citespace is used to show the citation trajectories of the top three authors in the field of SA. The three authors’ clustering and research fields are significantly different. The citations cited by Professor Zvolensky are mainly located in clusters #0, #9, #11, #14 (**Figure 12A**). It is worth noting that the citations cited by Professor Pine are mainly located in clusters #1, #3, #5, #9 (**Figure 12B**). The citations cited by Professor Heimberg are mainly located in clusters #0, #2, #4, #6, #9, #10, #12, #14, #15 (**Figure 12C**), which indicates that the author has great transformative potentials. The citation trajectories of these authors link several different research clusters, which not only helps to promote knowledge flow and connections within academic fields, but also advances cross-disciplinary academic interactions and collaborations.

## 4 Discussion

### 4.1 General information

To understand the research profile in the field of SA, we conducted a comprehensive analysis of the number of publications, authors, countries, institutions, and journals. The results show that the annual number of publications in the field of SA has been steadily increasing over the past decade, indicating that researchers’ interest in the field throws heat. And the literature comes from multiple disciplinary areas, mainly dominated by psychiatry, psychology and neurosciences. In terms of authors, there is a significant overlap between productive and influential authors in the field, with Zvolensky, Michael J (82,0.06), Pine, Daniel S (81,0.16), and Heimberg, Richard G (78,0.11), having the highest number of publications. The citation trajectories of the authors shows that Heimberg, Richard G’s research spans multiple clusters, which is a good indication of the outstanding contribution this author has made to research in the field of SA. In terms of national publication volume and top ten institutions, the United States has placed a great deal of emphasis on research in the field of SA. The top three journals in terms of publications are Frontiers in Psychology, Journal of Affective Disorders, and Journal of Anxiety Disorders. The top three co-cited journals are Behavior Research and Therapy, Journal of Anxiety Disorders, and Clinical Psychology Review.

### 4.2 Research hotspots

Combining the results of keyword co-occurrence analysis, cluster analysis and co-citation analysis can help us better understand the research hotspots in the field of SA.

First, the symptomatic manifestations of SA, especially cognitive impairment, are of concern. Keyword clusters #0 social cognition, #2 attentional bias, and #5 anxiety sensitivity emphasize abnormalities of cognitive functioning in SA (33). Attentional bias to threatening information has been shown to be a trigger and sustaining factor for SA, and this is supported by cognitive-behavioral models of SA (34). Eye tracking technology (#6) provides objective metrics that are also commonly used to measure deficits in facial expression recognition (#13), attentional bias, and other deficits in socially anxious individuals.

Second, risk factors that trigger SA have also been hot topics of research, including comorbidities, personal traits, and peer factors. Underlying personal traits as well as comorbidities can aggravate an individual’s risk for SA, such as: keyword clusters #12 behavioral inhibition (35), #4 anxiety disorders (36), #9 autism spectrum disorder (37), and #10 eating disorder (38). Up to 90% of socially anxious people experience co-morbidities, and overlapping symptoms are not only easy to misdiagnose, but also increase the risk of suicide. Peer relationships play an important role in individual development. Peer victimization (#17) can cause adolescents to develop negative perceptions, and repeating this experience can continually deepen adolescents’ negative perceptions of social activities, which also decreases cognitive flexibility and avoidance of social activities, which in turn increases levels of SA (39). Meanwhile, the negative effects of SA have received equal attention, such as: decreased #14 quality of life (40), #16 internet gaming disorder (41), and #20 substance use (42).

Third, neuroimaging studies of SA are also important. #11prefrontal cortex is involved in the neurophysiological basis of SA. Tian et al. identified a key role of the prefrontal temporal circuit in the regulation of social fear (43). The top 20 citing articles in co-citation cluster #0 also emphasize the integration of neuroimaging with cognitive mechanisms, intervention strategies, and neurobiological studies. This branch of research may contribute to the development of personalized treatment or precision medicine for SA.

Finally, intervention strategies for SA are hot topics. For example, the keyword clusters #3, #8 as well as the citing literature on the effects and predictors of therapies in cluster #3 of the co-citation analysis can provide strong support for this hot topic. In particular, cognitive behavioral therapy has gained the attention of many researchers. Cognitive behavior therapy (#8) is a very safe and effective approach to intervene in SA (44), with threatening perceptions of social situations being the focus of important interventions, and combined with exposure therapy (#3), where the individual needs to be confronted with fearful or anxious situations (45). In addition, the ability of emotional regulation (#18) is crucial. Regulating an individual's emotions during the cognitive behavioral therapy intervention process helps to enhance the effectiveness of the intervention (46). Social skills (#19) training is an essential component of evidence-based treatment guidelines. When socially anxious individuals acquire appropriate social skills, they will be better able to cope with various social situations, which will lead to more positive and emotional experiences in social situations. This will help in building various interpersonal relationships and also deep belief revision (47). Among the various interventions for SA, cognitive-behavioral therapy is significantly better than other psychotherapies. However, cognitive-behavioral therapies focus primarily on thought patterns and behavioral habits and may show limited effectiveness in addressing non-cognitive factors contributing to psychological problems. Given that etiological models of SA emphasize that genetics, temperament, environment, and cognition interact to increase the risk for the development of SA, cognitive-behavioral therapy, in combination with other psychotherapeutic approaches (e.g., psychodynamic therapy, humanistic orientation therapy, etc.), may enhance therapeutic efficacy. In addition, with the development of technology, cognitive behavioral therapy can also leverage new technologies (e.g., the Internet, artificial intelligence) to achieve remote and intelligent treatment, improving accessibility and convenience.

### 4.3 Research frontiers

Keyword burst analysis can reflect the frontiers during a specific period of time in the research field, helping scholars predict research trend and grasp the research direction. The following frontier topics were identified through the burst analysis: “theory of mind”, “bullying victimization”, “mobile phone”, “network analysis”, “technology”, and “satisfaction”.

First, the recent burst in the term “theory of mind” indicates that theory of mind is at the forefront of the SA field. The key for harmonious socializing and interpersonal interaction is having a “theory of mind” (ToM), or the ability to accurately decode and reason about the beliefs, intentions, desires, and emotions of others (48). A cross-sectional study of adolescents by Yusuf Ozturk et al. showed a negative association between the severity of SA symptoms and the ToM task (Reading the Mind in the Eyes test, RMET). However, the RMET measured only the decoding part of ToM, focusing on emotion and ignoring the more complex reasoning component (49). Another longitudinal study (50) showed that children with low ToM ability had an indirect effect on SA from 2 years of age (behavioral inhibition, BI) to 4.5 years of age BI to 6.5 years of age, while children with high ToM ability had no indirect effect. In addition, this study also found that there is a curvilinear relationship between ToM and SA, that is, for children with low ToM ability, ToM is negatively correlated with SA, while for children with high ToM ability, there is no correlation between the two. This study used the false belief task to measure children’s ToM, focusing on the relationship between cognitive aspects of ToM and SA (50). Studies conducted with children aged 8-12 years have found that there is a conic relationship between ToM ability and SA, i.e. children with lower and higher ToM ability both show higher levels of SA. The study used RMET to measure ToM (51). In summary, we can find that the relationship between SA and ToM is complex and may be related to the research paradigm, the age of the subjects, and other factors that need to be further explored in future studies. In addition, future research may be able to combine the task of assessing different components of ToM, such as cognitive and affective components, decoding and reasoning processes, to obtain a more systematic and comprehensive understanding of the impact of an individual’s ToM ability on psychopathology, thus providing a more reliable basis for prediction and intervention.

Second, the problem of bullying (cyberbullying and traditional bullying) is becoming more and more serious, especially the negative impact on individuals’ SA, which has attracted increasing attention from researchers, as evidenced by the burst of the term “bullying victimization”. Repeated physical attacks, verbal taunts, relational ostracism and even spreading rumors on the Internet by a bully over a long period can directly cause great pain and fear to victims. They may choose to isolate themselves from others to avoid re-victimization. Studies have found that bullies, cyber and traditional bully-victims showed higher levels of SA than uninvolved students (52). Over time, the increase in SA for victims and bullying-victims will be greater (53). Cañas et al. found that cyberbullies had significantly higher anxiety due to fear of negative evaluation from others (54). However, how long an intervention they need to gradually alleviate the uncomfortable symptoms of SA may be worth exploring further in the future. Martínez-Monteagudo used latent category analysis to classify cyberbullying into three categories: high cyberbullying (high bully, high victim, high bully-victim), low cyberbullying, and non-cyberbullying, and found that students who were high cyberbullying (i.e., bully-victims) exhibited higher levels of social avoidance and social distress in social situations with peers (55). Overall, it seems that the underlying variables between bullying and SA still need to be further explored in the future, and the role played by individuals’ positive psychological qualities or externally controllable factors could be further examined subsequently. Cross-sectional designs cannot establish causal relationships between variables or determine the long-term effects of SA on bullied individuals. Therefore, conducting more longitudinal or intervention studies is recommended to better address these issues. Additionally, bystanders/non-participants may be a neglected group in current research, displaying considerable heterogeneity, and measuring and addressing this issue may provide a theoretical basis for the development of bystander interventions.

Third, the burst of the term “mobile phone” suggests that research related to SA and mobile phone addiction is becoming a cutting-edge topic. Mobile phone addiction refers to a kind of behavioral addiction due to an individual’s uncontrollable use of mobile phones, leading to significant impairment in physiological, psychological, and social functions (56). The majority of studies support SA as a risk factor for mobile phone addiction (57–59), and most of such studies have examined the relationship between mobile phone addiction and SA in mediation or moderation models using cross-sectional research designs. However, few studies have explored directly examining important mediating and moderating variables that influence the association between SA and mobile phone addiction. The directionality of this link remains an area of interest. Whether mobile phone addiction affects SA is still an open question. A recent longitudinal study found a causally predictive relationship between SA and mobile phone addiction among college students, with SA acting as both an antecedent and a consequence of mobile phone addiction (60). However, this study only collected data at two time points. Future research should use longitudinal designs with multiple time points to investigate the dynamic nature of the relationship between mobile phone addiction and SA, and explore the possibility of a vicious cycle by constructing more comprehensive causal models. In addition, combining the experimental design to establish the causal relationship will greatly deepen our understanding of the relationship, for example, to further explore the motivation, way, and type of individual’s mobile phone use behaviors, as different mobile phone use behaviors may be associated with SA in different ways. This will enable researchers to further explain the relationship between mobile phone addiction and SA.

Fourth, “network analysis” is also a keyword that has burst in recent years. Network analysis places the symptoms of mental illness in an interconnected network, describes the relationships between them, and identifies the core symptoms of the illness based on the centrality of the network. Using network analysis methods to study the core symptoms of psychiatric disorders and targeting them for intervention can enhance the effectiveness of intervention and has great practical value. Currently, network analysis has made some progress in the field of SA. For example, network analyses showed that feeling ridiculed, fear of rejection, difficulty asking others to do things together, and feeling left out at school were important bridge symptoms for the comorbidity of loneliness and SA (61). Cognitive dysfunction plays a central role in the SA symptom network (62). Additionally, feelings of worthlessness and anhedonia are key symptoms in the comorbidity network of major depression and SA (63). Most network analyses use cross-sectional data, which means that it is difficult to reflect changes in the time dimension, and later researchers can use longitudinal research methods. Furthermore, after identifying core symptoms through network analysis, the validity of these results can be tested in the future by intervening through clinical control.

Fifth, the burst in the term “technology” may be attributed to the development of digital intervention technologies in the field of SA, e.g., mindfulness-based mobile apps (64), online cognitive therapy (65), and VR/AR exposure therapy (66). It is also related to the introduction of research methods, such as cue word technology (67) and event-related potential technology (68).

Finally, the burst in the term “satisfaction” may be related to variables related to SA, such as satisfaction with physical appearance (69), life satisfaction (70), and basic psychological needs satisfaction (71). It may also be linked to the appearance of intervention-related keywords, such as treatment satisfaction (72).

### 4.4 Future research prospects

To summarize, considerable research results have been achieved in the field of SA over the past decade, but further in-depth discussions can still be conducted in the following two aspects.

On the one hand, we should promote multidisciplinary cross-collaboration in SA research and enhance the diversity of research methods. In the future, we can expand the distribution of SA research disciplines and strengthen the communication and cooperation between researchers and organizations in different disciplines, so as to coordinate the research methods within each field. Most of the current studies used self-report to measure SA, and future studies could adopt a more integrated approach to data collection using multiple instruments (e.g., observation, questionnaires, and interviews), multiple channels (e.g., self-report, teacher-report, parent-report, and peer-report), and objective assessments (e.g., autonomic nervous system responses). As Fernández et al. demonstrated, thermal changes in the tip of the nose may be a physiological indicator of SA (73). In addition, with the help of cognitive neuroscience tools and techniques to open the “black box” of the brain. For example, Mizzi et al. found that SA severity was significantly negatively correlated with functional connectivity of the precuneus - perigenual anterior cingulate cortex and positively correlated with functional connectivity of the amygdala (specifically the superficial subregion) - parietal/cerebellar areas (74). Finally, and equally importantly, multimodal research can provide a more comprehensive understanding of the complexity of SA, occupying higher research perspectives through the intervention of multiple technological tools, thus obtaining more accurate and insightful findings (75).

On the other hand, we need to pay attention to the assessment, diagnosis, and intervention of SA comorbidity. The results of this study show that the comorbidity of SA is a hot topic of research. Comorbidities may impair patients’ symptoms through multiple mechanisms, exacerbating their impact on work efficiency, family relationships, and quality of life, while also increasing the difficulty of diagnosis and treatment (76, 77). In the future, we should further explore the related comorbidities and conduct in-depth research on the symptomatic association between these disorders. For example, network analysis can be used to explain the relationship between the symptoms of different psychological disorders, and to explore the association between symptoms and the potential mechanism of interaction through network analysis. In addition, SA intervention research is also a hot topic. However, most of the interventions have been targeted at people with SA, and there is still limited research on interventions for other psychiatric disorders that are co-morbid with SA. Baljé et al. demonstrated the effectiveness of schema therapy and cognitive behavioral therapy in improving SA comorbid with avoidant personality disorder in a randomized controlled trial (78), and Pavlova et al. found that cognitive behavioral therapy is an acceptable, safe, and effective treatment for SA in patients with bipolar disorder (79). Further research should be conducted in the future in order to discover more effective and individualized treatments.

In conclusion, the factors and mechanisms that induce SA ought to be studied from a multidisciplinary, multilevel, and multifaceted perspective. Basic research has to be combined with clinical research, and systematic and in-depth research should be carried out at the three levels of prevention, diagnosis, and treatment. Early detection, early diagnosis, early treatment, and optimization of clinical treatment of SA should be achieved to improve the efficiency and prognosis of clinical treatment of SA.

## 5 Limitations

Although our study provides a degree of thought and inspiration for research in the field of SA, some of the limitations should be considered. First, the data source for this study is only one database, WoS, which leads to the possible omission of some other relevant literature. In the future, multiple databases can be considered for searching or comparative analysis. Second, while the software used in this study provides objective results, the interpretation and analysis of the findings involve a degree of subjectivity. Finally, this study only uses one bibliometric analysis tool, and future studies can use different analysis tools to analyze more data.

## 6 Conclusion

In this study, we used CiteSpace software to visualize and analyze the SA research literature over the last decade, including authors, countries, institutions, journals, keywords, and references aiming to get a comprehensive picture of its development and trends. The results emphasize that Zvolensky, Michael J has the highest number of publications in the field, and Heimberg, Richard G has a broader research orientation. The United States has the largest number of publications, with the University of California System contributing the most. The research hotspots in the field focus on intervention strategies, cognitive impairment, risk factors, comorbid conditions, and neuroimaging. Theory of mind, network analysis, technology, satisfaction, bullying victimization, and mobile phone are research frontiers. Future studies should further promote multidisciplinary cross-collaboration in SA research, implement diverse research methods and emphasize the assessment and diagnosis of comorbidities. Most importantly, early diagnosis and treatment are essential for alleviating SA, which requires increased public awareness of its causes and dangers. Therefore, it is crucial to raise awareness of the public health implications of SA.
